# Trends of pulmonary fungal infections from 2013 to 2019: an AI-based real-world observational study in Guangzhou, China

**DOI:** 10.1080/22221751.2021.1894902

**Published:** 2021-03-13

**Authors:** Zhengtu Li, Yongming Li, Yijun Chen, Jing Li, Shaoqiang Li, Chenglong Li, Ye Lin, Wenhua Jian, Jingrong Shi, Yangqing Zhan, Jing Cheng, Jingping Zheng, Nanshan Zhong, Feng Ye

**Affiliations:** a State Key Laboratory of Respiratory Disease, National Clinical Research Center for Respiratory Disease, Guangzhou Institute of Respiratory Health, the First Affiliated Hospital of Guangzhou Medical University, Guangzhou, People’s Republic of China; b Guangzhou Tianpeng Technology Co., Ltd., Guangzhou, People’s Republic of China

**Keywords:** Epidemiology, incidence, trends, artificial intelligence, pulmonary fungal infection

## Abstract

Recently, the prevalence trend of pulmonary fungal infection (PFI) has rapidly increased. Changes in the risk factors for, distributions of underlying diseases associated with and clinical characteristics of some individual PFIs have been reported in the past decade. However, data regarding PFIs remain uncertain. This study reports the epidemiological characteristics and trends of PFIs over time in recent years. We applied an automated natural language processing (NLP) system to extract clinically relevant information from the electronic health records (EHRs) of PFI patients at the First Affiliated Hospital of Guangzhou Medical University. Then, a trend analysis was performed. From January 1, 2013, to December 31, 2019, 40,504 inpatients and 219,414 outpatients with respiratory diseases were screened, in which 1368 inpatients and 1313 outpatients with PFI were identified. These patients were from throughout the country, but most patients were from southern China. Upward trends in PFIs were observed in both hospitalized patients and outpatients (*P*<0.05). The stratification by age showed that the incidence of hospitalized patients aged 14–30 years exhibited the most obvious upward trend, increasing from 9.5 per 1000 patients in 2013 to 88.3 per 1000 patients in 2019. Aspergillosis (56.69%) was the most common PFI, but notably, the incidence rates of *Talaromyces marneffei*, which used to be considered uncommon, exhibited the most rapid increases. In younger PFI patients, the incidence and trend of PFIs have increased. Infection by previously uncommon pathogens has also gradually increased. Increased attention should be paid to young PFI patients and uncommon PFI pathogen infections.

## Introduction

With the widespread use of antibiotics, glucocorticoids, and immunosuppressive agents, and the growing population of immunocompromised patients, such as cancer and HIV patients, the incidence of pulmonary fungal infections has increased [1,2]. PFIs used to occur mainly in HIV and other immunocompromised patients, but an increasing series of reports indicates that immunocompetent and immunocompromised patients without traditional risk factors are affected [3–7]. Recently, accumulating evidence of variations in risk factors, distributions of underlying diseases and basic clinical characteristics associated with aspergillosis, cryptococcosis and *Talaromyces marneffei* have been reported [4–7]. However, among pulmonary fungal diseases, whether the traditional clinical characteristics have changed remains uncertain. Studies with substantial data in this research area are urgently needed to provide deeper insight into pulmonary fungal infections and guide clinical physicians.

In medicine, artificial intelligence (AI) methods have emerged as potentially powerful tools for mining electronic health record data to aid in disease diagnosis and management [8], but the analysis of EHR data presents several challenges, including the substantial amount of data and deviations or systematic errors in medical data [9]. However, a recent study reported that AI could overcome this difficult challenge through an automated NLP system and achieved excellent results [10].

Therefore, given the limited knowledge of changes in PFIs and the superiority of AI in analysing a substantial amount of data, we retrospectively extracted clinically relevant information from the EHRs of PFI patients using an automated NLP system. The aim was to investigate changes in the epidemiological characteristics of PFI patients.

## Materials and methods

### Ethics statement

This study was approved by the ethics committee of the First Affiliated Hospital of Guangzhou Medical University (Ethical number: 2018-119). This study was a retrospective case series study, and no patients were involved in the study design, setting the research questions, or the direct outcome measures. No patients were asked for advice regarding the interpretation or reporting of results.

### Study design and participants

We applied an automated NLP system to retrospectively select all patients with confirmed PFIs between 2013 and 2019 at the Department of Respiratory Medicine of the First Affiliated Hospital of Guangzhou Medical University. This department is famous for respiratory medicine in China and has been the top respiratory medical centre since 2009, with the average annual number of outpatient visits reaching over 117,146 and the average number of hospitalizations reaching 76,217 over the past 7 years.

**Figure 1.**
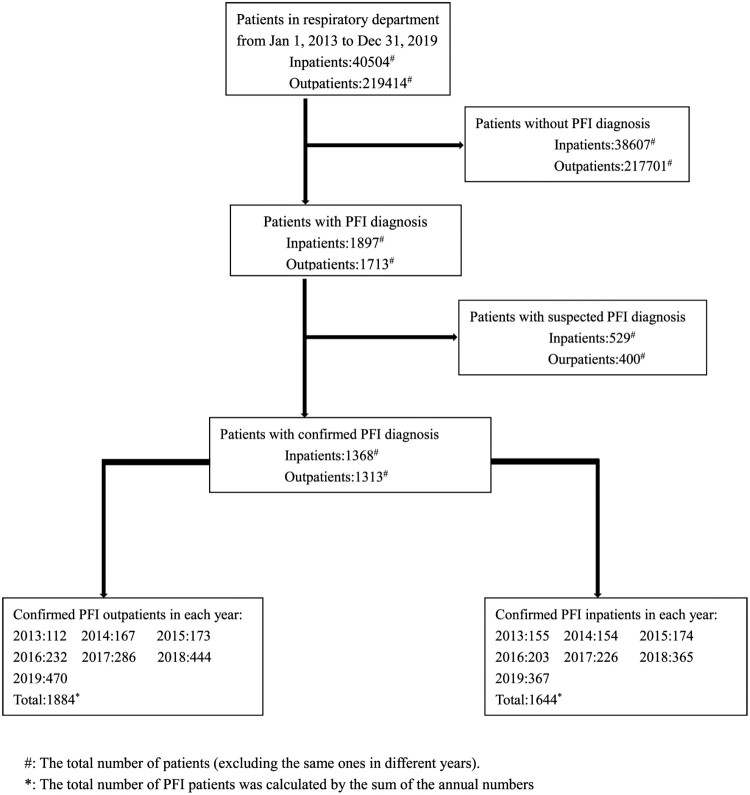
Study profile. *The total number of PFI patients was calculated by the sum of the annual numbers. ^#^The total number of patients (excluding the same ones in different years).

First, based on diagnosis information from patients’ medical records, AI comprehensively distinguished between patients with and without PFIs. Subsequently, PFI patients with suspected diagnoses were excluded (Figure 1). Then, the clinical information (including sex, age, pathogens and underlying diseases), admission costs, drug use status and adverse events were extracted from the patients’ records. After data collection, trend analyses of the changes in the characteristics of the PFI patients in recent years was performed. Multiple admissions in different years were considered separately, and missing data were excluded from the analysis.

### Data production process

The data analysed in this study were mainly produced by the following process. All data acquisition was authorized by the relevant departments of the hospital. By docking with the hospital integrated platform or clinical business system, the data extraction was performed during specified non-business hours of the hospital by means of “push view, intermediate library, message bus, and webservice” to collect the historical data and incremental data of outpatients and inpatients.

After the completion of the data collection, we analysed the EHRs according to the recognized guidelines [11,12] and basic dataset of electronic medical record (sourced from the National Health Commission (http://www.nhc.gov.cn/)) and established a series of data application standards, including standard medical terms and synonyms, etc. In addition to the application of poststructural technology, the collected data were deep-cleaned and verified before forming a standardized specialized disease database, and then the data application was analysed.

During the data collection process, we strictly followed data security standards [13]. To ensure the safety of the data throughout the process, the construction of the platform met the criteria of the national “three levels of information security protection” certification and “ISO27001” certification.

**Figure 2.**
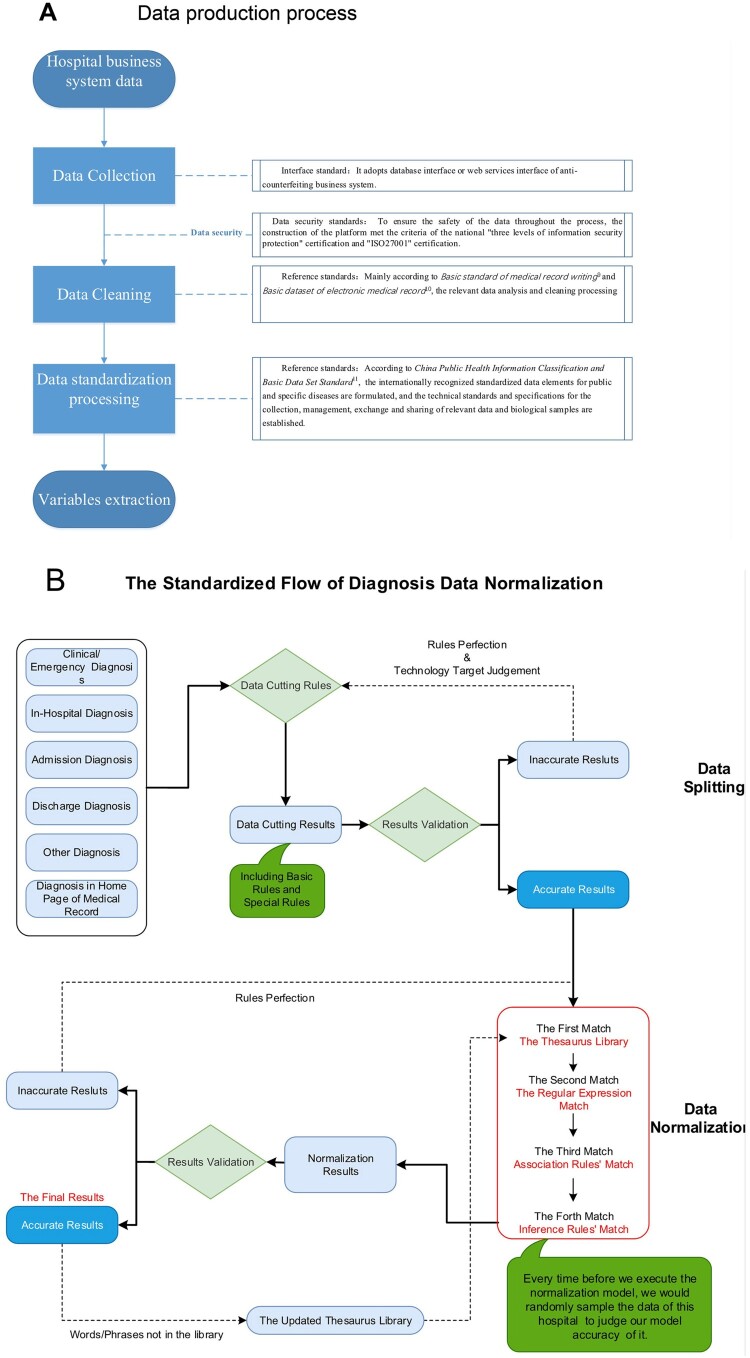
Data processing. Data production process (A), Data standardization process (B).

Regarding the accuracy, we conducted in-depth data verification considering the data acquisition, data processing and data production and produced a data verification report covering the whole process of the data production to ensure the accuracy, authenticity and reliability of the platform output data (Figure 2(A)).

### Data standardization process

First, a diagnostic standard terminology database was established according to the International Classification of Diseases Volume 10 (ICD-10) and Systematized Nomenclature of Medicine Clinical Terms (SNOMED) standard terminology. Then, by adding clinical case data, we created a synonym database to achieve accurate mapping between the original diagnosis data and standard terminology. The diagnostic standardization process mainly included the following four steps: diagnostic parsing, diagnostic splitting, diagnostic matching, and diagnostic validation.

(1). Diagnostic parsing: Diagnostic data from the medical record homepage, outpatient medical records and discharge records were parsed to generate original diagnostic data.
(2). Diagnosis splitting: In cases with multiple disease diagnoses, the data were split to generate a single original diagnostic diagnosis. If the results were inaccurate in the validation, the rules were improved, and the erroneous results were split according to modified rules until accurate results were obtained.
(3). Diagnosis matching: After splitting, the original diagnostic data were matched by identifying existing synonyms in the diagnostic database. If no synonym was matched, the split raw data were normalized manually and then mapped to the standard diagnostic terminology.
(4). Diagnostic validation: The raw diagnostic data after normalization were manually checked through random sampling and then released after validation. Accurate results were added to the updated thesaurus library when the words or phrases were not found in the library. Inaccurate results were normalized again according to new rules (Figure 2(B)).

### Statistical analysis

The primary objectives of this research were to study the trend of PFI by age, pathogens and underlying diseases in recent years and the secondary objectives included analysing the trend in sex, incidence of patients with adverse events, mean length of hospital stay, admission costs and drug proportion. A trend analysis was performed using the proportions of PFI patients among all patients with respiratory diseases. The percentage change in the annual incidence and 95% CI were estimated using a linear regression analysis of the log of the annual incidence. The data were analysed using SPSS (IBM SPSS Statistics 27, SPSS, Inc, Chicago, USA) and R (version 4.0.2). All statistical tests were bilateral tests, and a *P*-value < 0.05 was considered statistically significant.

## Results

### Epidemiological features of PFI patients

**Figure 3.**
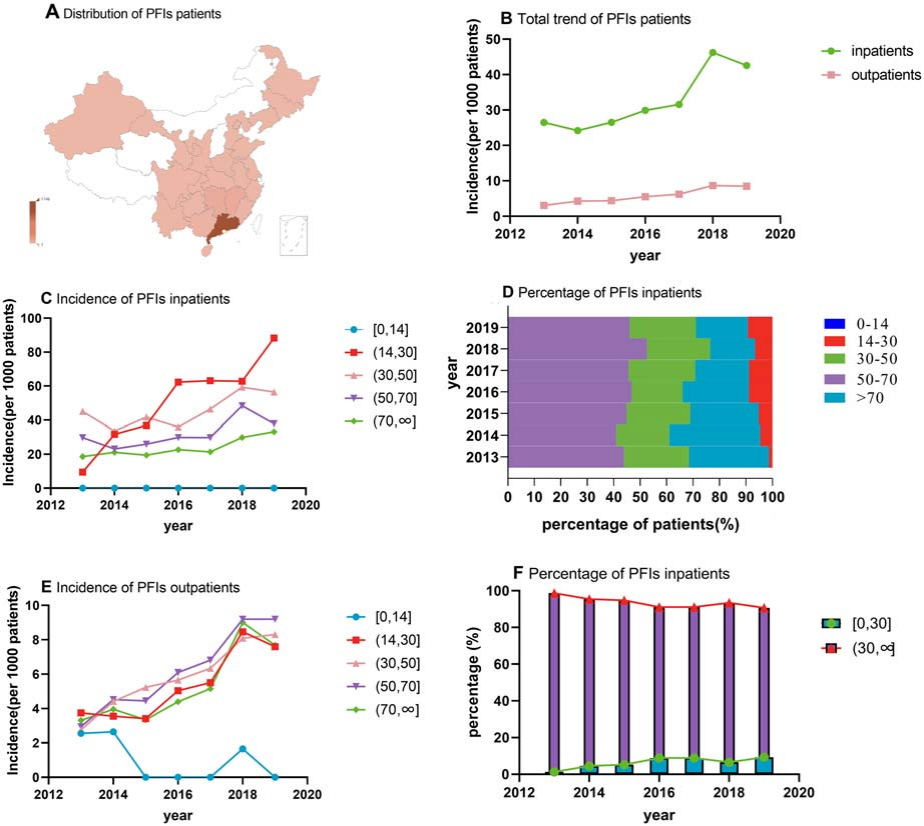
Distribution of PFI patients and trends of the incidence of PFI patients by age. Distribution of patients in this study (A), incidence of all PFI patients (B), incidence of inpatients in different age groups (C), percentage of PFI inpatients (D), incidence of PFI outpatients (E), and percentage of PFI inpatients in the <30 age group and >30 age group (F).

Overall, 1368 inpatients and 1313 outpatients at the Department of Respiratory Medicine of our hospital were identified between 2013 and 2019. These patients were from throughout the country, but most patients were from Guangdong [1146 (80%)], Jiangxi [61 (4.3%)], Hunan [55 (3.9%)] and Guangxi [31 (2.2%)] (Figure 3(A)). This distribution could be attributed to the more humid environment and more convenient transportation than those in other provinces. Some basic clinical data of the subjects in this study were lost, and missing values were not included in the analysis. Thus, the number of subjects included in the analysis differ from the number enrolled. The stratification by age showed that the patients in the age group of 50–70 years comprised the largest proportion of the study population [inpatients: 46.72%; outpatients: 42.41%], followed by those in the age groups of 30–50 years [inpatients: 23.48%; outpatients: 33.65%], >70 years [inpatients: 22.87%; outpatients: 10.93%], 14–30 years [inpatients: 6.93%; outpatients: 12.85%] and <14 years [inpatients: 0%; outpatients: 0.16%]. Males [inpatients: 70%; outpatients: 64.21%] were more likely to suffer from fungal infections than females. Pulmonary aspergillosis [56.69% (*n*=932)] and cryptococcosis [17.03% (*n*=280)] occurred in most hospitalized patients, and most patients had one or more underlying diseases (Supplemental material eTable1 and 2).

### General trends of PFI

Among hospitalized patients, the number of PFI patients significantly increased from 155 in 2013 to 367 in 2019. Among all patients admitted for respiratory diseases, the proportion of PFI patients increased annually from 26.5 per 1000 patients in 2013 to 42.6 per 1000 patients in 2019, with the highest incidence of 46.2 per 1000 patients in 2018. Among outpatients, the trend was similar. The incidence of PFI patients seen in the outpatient department increased yearly from 3.07 per 1000 patients in 2013 to 8.48 per 1000 patients in 2019 (Figure 3(B)).

### Trends by age

Most PFI patients, including both hospitalized patients [46.72% (*n*=768)] and outpatients [42.41% (*n*=799)], were in the 50–70 age group. The number of outpatients in the 14–30 age group exceeded the number of outpatients in the over 70 age group, while the opposite result was observed among hospitalized patients. Among these patients, the incidence of hospitalizations for PFIs in the 14–30 age group and the over 70 age group showed increasing annual linear trends (*P*<0.05). Among them, the incidence of hospitalized patients with fungal infections in the 14–30 age group showed a more obvious increasing trend than those in the other age groups, increasing from 9.48 per 1000 patients in 2013 to 88.31 per 1000 patients in 2019. Considering the age of 30 years as the cutoff, we found that among the hospitalized patients, the percentage change in the annual incidence in the 0–30 age group was much greater than that in the over 30 age group (13.2% vs 4.1%) and that the proportion of younger patients increased, while the proportion of older patients decreased. Similarly, among the outpatients, an upward trend was also observed in the 14–30 age group (*P*<0.05), with a 6.7% percentage year-on-year increase Although the annual percentage change was greater in the older patient group, the difference was very small (6.2% vs 7.8%). Nevertheless, an overall increasing trend was observed among the younger PFI patients (Figure 3, Table 1).

**Table 1.** Incidence of PFI patients by age and sex.

			Annualincidence					Percentage change inthe annual incidence(95% Cl)*	*P* value
	2013	2014	2015	2016	2017	2018	2019		
Total trend									
Inpatients	26.5 (155)	24.2(154)	26.5(174)	29.9(203)	31.6(226)	46.2(365)	42.4(367)	4.5(2.0–7.0)	0.006
Outpatients	3.1(112)	4.2(167)	4.4(173)	5.5(232)	6.2(286)	8.6(444)	8.4(470)	7.5(5.6–9.3)	<0.001
Sex (inpatients)									
Male	28.9(106)	25.7(105)	29.1(123)	34.3(149)	35.3(164)	49.4(252)	44.4(243)	4.4 (2.1–6.7)	0.005
Female	22.5(49)	21.5(49)	21.9(51)	22.0(54)	24.7(62)	40.4(113)	35.6(112)	4.3(0.7–7.9)	0.027
Sex (outpatients)									
Male	3.6(65)	5.2(102)	5.6(110)	7.1(149)	8.0(185)	11.1(287)	10.8(302)	7.9(5.9–10.0)	<0.001
Female	2.5(46)	3.3(64)	3.2(63)	3.9(82)	4.4(99)	6.0(152)	5.9(163)	6.4(4.6–8.2)	<0.001
Age (inpatients)									
[0,14]	0.00(0)	0.00(0)	0.00(0)	0.00(0)	0.00(0)	0.00(0)	0.00(0)	NA	NA
(14,30)	9.5(2)	31.7(7)	36.9(9)	62.3(18)	63.0(20)	62.8(24)	88.3(34)	13.3(5.4–21.3)	0.008
(30,50)	45.1(38)	33.7(31)	41.7(42)	36.0(39)	46.5(57)	59.3(87)	56.4(92)	3.0(−0.5–6.5)	0.081
(50,70)	29.8(68)	23.1(63)	26.0(78)	29.9(95)	29.8(113)	48.4(192)	37.9(169)	3.6(−0.2–7.5)	0.06
(70,∞ )	18.7(47)	21.2(53)	19.5(45)	22.7(51)	21.4(46)	29.9(62)	33.2(72)	3.9(1.6–6.2)	0.008
Age (inpatients)									
[0,30]	9.5(2)	31.7(7)	36.9(9)	62.3(18)	62.1(20)	62.5%(24)	88.1%(34)	13.2(5.4–21.0)	0.007
(30,∞ )	27.1(153)	23.9(147)	26.1(165)	28.4(185)	30.1(206)	45.4(341)	40.3(333)	4.1(1.3–7.0)	0.014
Age (outpatients)									
[0,14]	2.6(1)	2.6(1)	0.00(0)	0.00(0)	0.00(0)	1.7(1)	0.00(0)	−4.2(−18.2–9.9)	0.165
(14,30)	3.7(24)	3.6(22)	3.4(20)	5.0(31)	5.5(35)	8.5(58)	7.6(52)	6.7(3.2–10.2)	0.004
(30,50)	2.8(36)	4.4(59)	5.2(73)	5.6(86)	6.3(97)	8.1(138)	8.3(154)	7.3(4.7–9.9)	0.001
(50,70)	2.9(37)	4.5(66)	4.4(67)	6.1(99)	6.8(125)	9.2(192)	9.2(213)	8.2(5.9–10.4)	<0.001
(70,∞ )	3.3(14)	4.0(19)	3.3(16)	4.4(22)	5.2(29)	9.0(55)	7.7(51)	7.1(3.2–11.1)	0.006
Age (outpatients)									
[0,30]	3.7(25)	3.5(23)	3.2(20)	4.6(31)	5.0(35)	7.9(59)	7.0(52)	6.2(2.3–10.0)	0.009
(30,∞ )	2.9(87)	4.4(144)	4.6(153)	5.7(201)	6.4(251)	8.7(385)	8.6(418)	7.8(5.8–9.8)	<0.001

Notes: The data represent the incidence per 1000 patients unless otherwise stated. * Estimated using a linear regression analysis of the log of the of the annual incide-nce (e.g. there was a statistically significant 5.9% year-on-year increase in the incidence of pulmonary aspergillosis during the 2013–2019 period).

### Trends by pathogens

**Figure 4.**
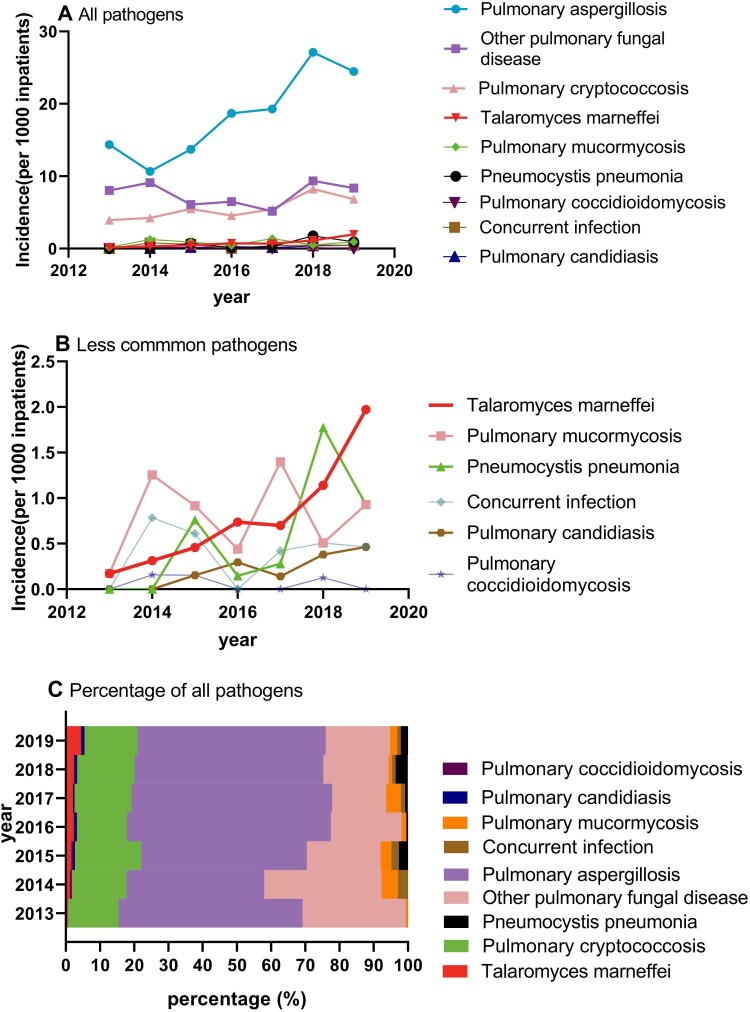
Trends of the incidence of PFI patients by pathogens. Incidence of all (A) and less uncommon (B) pathogens, and percentage of all pathogens (C).

The most common infection responsible for PFIs in the hospitalized patients was aspergillosis [56.69% (*n*=932)], followed by cryptococcosis [17.03% (*n*=280)] and *Talaromyces marneffei* [2.55% (*n*=42)]. Among them, the incidence of patients with aspergillosis, cryptococcosis, and *Talaromyces marneffei* showed upward trends (*P*<0.05). Interestingly, the incidence of *Talaromyces marneffei*, which is a strain that was previously uncommon, presented the fastest growth during the study period among all PFI patients from 0.17 per 1000 patients in 2013 to 1.97 per 1000 patients in 2019, representing a 16% percentage year-on-year increase (*P*<0.001). Other uncommon infections, such as pneumocystis pneumonia, pulmonary mucormycosis, and candidiasis, also presented a rapid increase but without a statistical significance, which could be related to the small samples. Additionally, in the internal percentage trend analysis of PFIs, we found that only the proportion of *Talaromyces marneffei* presented a rapid increase, from 0.65% to 4.63% (*P*<0.05), and other pathogens that were previously uncommon also experienced rapid increases but without statistical significance (Supplemental material eTable3). These findings indicate that the incidence of some infections that were previously uncommon has increased, especially since 2016, and additional attention should be paid to some PFIs that considered rare (Figure 4, Table 2).

**Table 2.** Incidence of PFI patients by infection.

Infection		Annual incidence						Percentage change in the annual incidence (95%Cl)*	*P* value
	2013	2014	2015	2016	2017	2018	2019		
Pulmonary aspergillosis	14.36(84)	10.67(68)	13.72(90)	18.70(127)	19.28(138)	27.10(214)	24.47(211)	5.9(2.3–9.5)	0.008
Other pulmonary fungal diseases	8.03(47)	9.10(58)	6.10(40)	6.48(44)	5.17(37)	9.37(74)	8.35(72)	0.02(−5.2–5.2)	0.994
Pulmonary cryptococccosis	3.93(23)	4.24(27)	5.49(36)	4.56(31)	5.45(39)	8.23(65)	6.84(59)	4.6(1.6–7.7)	0.011
*Talaromyces marneffei*	0.17(1)	0.31(2)	0.46(3)	0.74(5)	0.70(5)	1.14(9)	1.97(17)	16.0(12.4–19.6)	<0.001
Pulmonary mucormycosis	0.17(1)	1.26(8)	0.91(6)	0.44(3)	1.40(10)	0.51(4)	0.93(8)	5.7(−10.0–21.4)	0.393
Pneumocystis pneumonia	0.00(0)	0.00(0)	0.76(5)	0.15(1)	0.28(2)	1.77(14)	0.93(8)	12.5(−32.0–57.1)	0.437
Concurrent infection	0.00(0)	0.78(5)	0.61(4)	0.00(0)	0.42(3)	0.51(4)	0.46(4)	−4.3(−9.6–0.9)	0.077
Pulmonary candidiasis	0.00(0)	0.00(0)	0.15(1)	0.29(2)	0.14(1)	0.38(3)	0.46(4)	10.8(−7.9–29.5)	0.164
Pulmonary coccidioidomycosis	0.00(0)	0.16(1)	0.15(1)	0.00(0)	0.00(0)	0.13(1)	0.00(0)	−2.4(−6.0–1.2)	0.075

Notes: The data represent the incidence per 1000 patients unless otherwise stated. *Estimated using a linear regression analysis of the log of the annual incidence (e.g. there was a statistically significant 5.9% year-on-year increase in the incidence of pulmonary aspergillosis during the 2013–2019 period).

### Trends by underlying diseases

**Figure 5.**
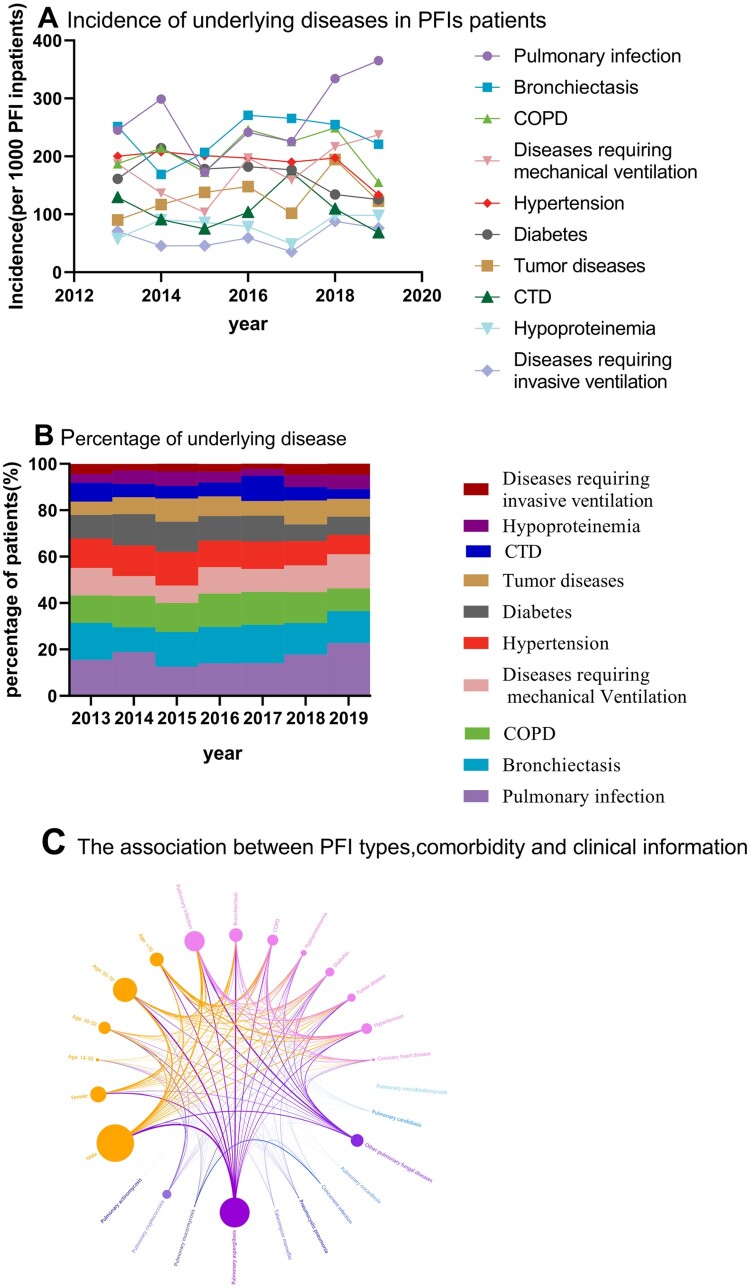
Trends of the incidence of PFI patients by underlying diseases. Incidence of underlying diseases in PFIs patients (A), percentages of underlying diseases (B), and the association between PFI types, comorbidity and clinical information (C).

Pulmonary infection (28.59%) was the most common underlying disease among the PFI patients, followed by bronchiectasis (23.72%) and chronic obstructive pulmonary disease (COPD) (20.74%). Although the incidence rates of all underlying diseases among the PFI patients remained stable in the recent 7 years, with no statistical significance, the increasing trend of pulmonary infection, bronchiectasis, diseases requiring mechanical ventilation, tumour diseases, hypoproteinemia and diseases requiring invasive ventilation were also observed (*P* >0.05). Here, we only showed the most common underlying diseases. For more details, see the supplement materials (eTable2). The analysis of relevance also showed that pulmonary infection and chronic pulmonary diseases may be strong independent risk factors for PFIs, especially pulmonary aspergillosis and cryptococcosis (Figure 5, Table 3). However, regarding uncommon pathogens, further research is still required.

**Table 3.** Incidence of PFI patients by underlying diseases.

Underlying Diseases			Annual incidence					Percentage change in the annual incidence (95% Cl)*	*P* value
	2013	2014	2015	2016	2017	2018	2019		
Pulmonary infection	24.52(38)	29.87(46)	17.24(30)	24.14(49)	22.57(51)	33.42(122)	36.51(134)	2.6(−2.5–7.7)	0.245
Bronchiectasis	25.16(39)	16.88(26)	20.69(36)	27.09(55)	26.55(60)	25.48(93)	22.07(81)	1.1(−2.7–4.8)	0.502
COPD	18.71(29)	21.43(33)	17.24(30)	24.63(50)	22.57(51)	24.93(91)	15.53(57)	0.02(−4.2–4.2)	0.99
Diseases requiring mechanical ventilation	18.71(29)	13.64(21)	10.34(18)	19.70%(40)	15.93(36)	21.64(79)	23.71(87)	3.2(−2.4–8.8)	0.198
Hypertension	20.00(31)	20.78(32)	20.11(35)	19.70(40)	19.03(43)	19.73(72)	13.35(49)	−2.1(−4.7–0.4)	0.085
Diabetes	16.13(25)	21.43(33)	17.82(31)	18.23(37)	17.70(40)	13.42(49)	12.53(46)	−2.6(−5.7–0.4)	0.077
Tumor diseases	9.03(14)	11.69(18)	13.79(24)	14.78(30)	10.18(23)	19.45(71)	12.26(45)	2.5(−2.5–7.6)	0.256
CTD	12.90(20)	9.09(14)	7.47(13)	10.34(21)	17.26(39)	10.96(40)	6.81(25)	−1.1(−8.4–6.2)	0.714
Hypoproteinemia	5.81(9)	9.09(14)	8.62(15)	7.88(16)	4.87(11)	9.86(36)	9.81(36)	1.8(−4.2–7.8)	0.473
Diseases requiring invasive ventilation	7.10(11)	4.55(7)	4.60(8)	5.91(12)	3.54(8)	8.77(32)	7.63(28)	2.0(−5.3–9.2)	0.515

Notes: The data represent the incidence per 100 PFI patients unless otherwise stated. *Estimated using a linear regression analysis of the log of the annual incidence. COPD: chronic obstructive pulmonary disease. CTD: connective tissue disease.

### Trends by adverse events and drug use

During the 2013–2019 period, the proportion of PFI patients who experienced adverse events (including death, the need for mechanical ventilation and intensive care unit admission) remained stable, with no significant upward or downward trend (Supplemental material eTable4). Although the number of hospitalizations increased annually, the mean length of hospital stays showed a fluctuating, decreasing trend from 13.54 days in 2013 to 9.48 days in 2019. An increasing fluctuating trend in admission costs was observed, but the variation was small, from 37,792.63 CNY (5835.18 USD) in 2013 to 38,406.16 CNY (5929.91 USD) in 2019. The proportion of patients receiving medicine also decreased annually (from 57.26% to 44.29%). Interestingly, the trends of broad-spectrum antibacterial and antifungal drug use were consistent with the fluctuation in antibacterial drug use (Supplemental material eFigure1).

## Discussion

This research aimed to use AI-based methods to study the epidemiological characteristics and trends in patients with pulmonary fungal diseases over time in recent years. Using an AI-based investigation, for the first time, we demonstrated a remarkable increasing incidence and trend from 2013 to 2019 in young PFI patients. The incidence of some PFIs that were previously considered rare or uncommon also rapidly increased. Standardizing diagnostic terms and applying AI in this study improved the quality of the data extraction. This 7-year real-world, big data study filled a gap in knowledge regarding the epidemiological characteristics of fungal disease, providing deeper insight into fungal infection and guidance for clinical physicians.

During the study period, a remarkable increasing incidence of PFI patients was observed. This sharp increase can be attributed to the increasing numbers of immunocompromised patients with malignancy, haematologic disease, and HIV, and those receiving immunosuppressive agents for organ transplantation or autoimmune inflammatory conditions [1,2]. In addition, PFIs were generally thought to occur in HIV and neutropenia patients. However, the number of reports documenting PFIs in immunocompetent patients or immunocompromised patients who do not have the classic risk factors is increasing [3–7]. Furthermore, advances in diagnostic methods and techniques have also greatly contributes to the identification of PFIs [1].

The stratification by age showed that older patients accounted for most PFI cases, and the incidence of PFIs continuously increased in this group. These findings are consistent with those reported in previous studies [14–16]. However, the proportion of younger patients with fungal diseases, especially those between the ages of 14 and 30, also increased annually. This result is comparable to previous findings in some individual pulmonary fungal diseases. The lung was the predominant site of invasive aspergillosis infection (83.4%), and 21.6% of the cases occurred in paediatric patients [15]. Although most cases of pulmonary aspergillosis occur in the 50–70 age group, younger patients aged under 30 also accounted for a considerable part [17]. In Colombia, younger patients (under 40) even accounted for approximately 59.26% of 1976 cryptococcosis patients [18].

However, the trend of PFIs in youth has not been reported in any previous studies. Here, we present strong evidence and are the first to report that the incidence of PFIs is increasing in younger patients. This finding may be related to the increased proportion of younger immunocompromised people [1,19]. Patients with high-risk factors are more likely to develop PFIs than patients without such factors [7]. Additionally, the discovery of novel immunodeficiency syndromes in children may contribute to the identification of additional at-risk patient groups [6]. Finally, advances in diagnostic methods and techniques, including the use of metagenomic next-generation sequencing (mNGS), computed tomography (CT), positron emission tomography (PET), and bronchoscopy, have also significantly contributed to the identification of PFI, thus increasing the PFI definitive diagnosis rate [1].

Pulmonary aspergillosis was found to be the most common PFI and exhibited a continuously upward trend, which is comparable to previous findings [20–22]. Importantly, the incidence of some pathogens that were previously considered uncommon also showed sharp increases, with *Talaromyces marneffei* infection showing the steepest upward trend (Figure 4). The development of highly active antiretroviral therapy and other effective control measures for the HIV/AIDS epidemic had resulted in the decreased incidence rate of *T. marneffei* infection in HIV patients.^23^ However, increasing cases have been reported in non-HIV-infected patients with anti-IFN γ autoantibodies and, those receiving immunosuppressive agents, such as anti-CD20 monoclonal antibodies and kinase inhibitors for malignancy, haematologic disease, organ transplantation and autoimmune inflammatory conditions [6]. Similar to the trend of *Talaromyces marneffei*, increasing trends of pulmonary mucormycosis, pneumocystis pneumonia and pulmonary candidiasis were also observed (*P* >0.05). In the future, additional cases of uncommon PFIs are likely to be reported in developing countries. The reason may be that improvement in the national health services in these countries likely lead to an increase in the population of non-HIV-infected patients at risk of infection, including transplantation recipients and cancer patients receiving targeted therapies [6]. Notably, we found that the incidence rates of these rare diseases had remarkably increased since 2016 compared to those in previous years. This finding may be a result of the application of mNGS technology [24,25] and the development of relevant guidelines [26]. Moreover, advances in diagnostic methods and techniques will greatly facilitate the detection and molecular characterization of causative pathogens [27–29].

Immunocompromised diseases (including malignancy, haematologic disease, and HIV infection) were previously the most common underlying diseases among fungal infection patients [20,22,30]. However, in this study, we found that pulmonary infections and chronic lung diseases may be great independent risk factors for PFIs, which is similar to the finding of recently published articles [15,21]. The risk in patients with these diseases is less severe than that in immunodeficient patients, but these diseases are more widespread and common and involve a much larger population than those causing immunodeficiency [31]. While the reason why PFIs are highly prevalent in lung infection patients remains unclear, evidence of this phenomenon has been reported. Influenza has been recognized as an independent risk factor for invasive pulmonary aspergillosis and is associated with high mortality [32]. Additionally, positive and negative interactions between Aspergillus and Pseudomonas aeruginosa, which are two central members of the fungal and bacterial pulmonary microbiota have also been reported [33,34]. Volatile compounds released by bacterial pathogens can stimulate the growth of fungal pathogens in lung infections [35]. In addition, chronic respiratory diseases, including COPD and bronchiectasis, have also been found to be great risk factors for PFIs [36,37].

PFIs are among the most common invasive fungal infections and present an increasing prevalence and serious threats to humans worldwide [15]. However, their importance is typically not well recognized or publicized. Most studies related to this topic were focused on invasive fungal infections [14,15,28,38] or individual PFI pathogens [4-6]. Knowledge regarding the epidemiology of PFIs in recent decades is relatively limited. To the best of our knowledge, this study is the largest and longest study ever performed investigating the trend in PFIs in recent years. These findings fill a gap in knowledge regarding the changed epidemiological characteristics of fungal disease, providing deeper insight into fungal infection and guidance for clinical physicians.

Limitations also exist in this research. First, this study was a single-centre study, and its conclusions may not apply to other countries. However, based on a large number of patients from throughout country and 7 years of real-world big data, we used NLP techniques to extract the data and then performed a trend analysis, thus ensuring that this single-centre analysis is representative. Second, this study was an observational study. Although some new insights into the clinical characteristics of PFIs have been reported, the causes of these trends and effective measures remain unknown. Our next study will explore the reason why a rapid increasing trend was observed among young PFIs patients.

In conclusion, the trend analysis revealed a remarkably increasing incidence and an increasing trend among younger PFI patients. The incidence of some PFIs previously considered rare or uncommon also showed rapid increases. These empirical findings in this study provide a new understanding of PFIs. Additional attention should be paid to young PFI patients and some previously uncommon PFIs that have shown a rapidly increasing trend.

## Supplementary Material

eFigure1.tif

eTable.docx

## Data Availability

The data supporting the findings of this study are available from the corresponding author upon reasonable request. Participant data without names and identifiers will be made available after approval from the corresponding author. After the publication of the study findings, the data will be available to other researchers upon request. The research team will provide an email address for communication once the data are approved for sharing with others. A proposal containing a detailed description of the study objectives and statistical analysis plan will be needed to evaluate the reasonability of the data request. The corresponding author will make a decision based on these materials. Additional materials may also be required during the process.

## References

[1] Limper AH, Knox KS, Sarosi GA, et al. An official American Thoracic Society statement: treatment of fungal infections in adult pulmonary and critical care patients. Am J Respir Crit Care Med. 2011;183(1):96–128. 21193785 10.1164/rccm.2008-740ST

[2] Limper AH. The changing spectrum of fungal infections in pulmonary and critical care practice: clinical approach to diagnosis. Proc Am Thorac Soc. 2010 May;7(3):163–168. 20463243 10.1513/pats.200906-049AL

[3] Denning DW, Chakrabarti A. Pulmonary and sinus fungal diseases in non-immunocompromised patients. Lancet Infect Dis. 2017;17(11):e357–e366. 28774699 10.1016/S1473-3099(17)30309-2

[4] Latgé J-P, Chamilos G. Aspergillus fumigatus and Aspergillosis in 2019. Clin AspergMicrobiol Rev. 2019;33(1):e00140–18. 10.1128/CMR.00140-18 PMC6860006 31722890

[5] O’Halloran JA, Powderly WG, Spec A. Cryptococcosis today: it is not all about HIV infection. Curr Clin Microbiol Rep. 2017;4(2):88–95. 29130027 10.1007/s40588-017-0064-8 PMC5677188

[6] Chan JFW, Lau SKP, Yuen K-Y, et al. Talaromyces (Penicillium) marneffei infection in non-HIV-infected patients. Emerg Microbes Infect. 2016;5:e19. 26956447 10.1038/emi.2016.18 PMC4820671

[7] Smith JA, Kauffman CA. Pulmonary fungal infections. Respirology. 2012;17(6):913–926. 22335254 10.1111/j.1440-1843.2012.02150.x

[8] Hu J, Perer A, Wang F, et al. Data driven analytics for personalized healthcare. In: Weaver CA, Ball MJ, Kim GR, editors. Healthcare information management systems: cases, strategies, and solutions. Cham: Springer International Publishing; 2016. p. 529–554.

[9] Turchin A, Kolatkar NS, Grant RW, et al. Using regular expressions to abstract blood pressure and treatment intensification information from the text of physician notes. J Am Med Inform Assoc. 2006;13(6):691–695. 16929043 10.1197/jamia.M2078 PMC1656954

[10] Liang H, Tsui BY, Ni H, et al. Evaluation and accurate diagnoses of pediatric diseases using artificial intelligence. Nat Med. 2019;25(3):433–438. 30742121 10.1038/s41591-018-0335-9

[11] NHC. Basic standard of medical record writing [5 April 2020]. Available from: http://www.nhc.gov.cn/yzygj/s3585u/201002/0517a82e35224ee0912a5d855a9d249f.shtml.

[12] NHC. China public health information classification and basic data set standard [5 April 2020]. Available from: http://www.nhc.gov.cn/mohwsbwstjxxzx/s8553/200809/37854.shtml.

[13] NHC. National Health and medical big data standards, safety and service management measures (Trial) [5 April 2020]. Available from: http://www.nhc.gov.cn/guihuaxxs/s10741/201809/758ec2f510c74683b9c4ab4ffbe46557.shtml.

[14] Zilberberg MD, Nathanson BH, Harrington R, et al. Epidemiology and outcomes of hospitalizations with invasive aspergillosis in the United States, 2009-2013. Clin Infect Dis. 2018 Sep 1;67(5):727–735. 29718296 10.1093/cid/ciy181 PMC7190884

[15] Webb BJ, Ferraro JP, Rea S, et al. Epidemiology and clinical features of invasive fungal infection in a US Health care network. Open Forum Infect Dis. 2018;5(8):ofy187. 30151412 10.1093/ofid/ofy187 PMC6104777

[16] Shaheen AAM, Somayaji R, Myers R, et al. Epidemiology and trends of cryptococcosis in the United States from 2000 to 2007: a population-based study. Int J STD AIDS. 2018 Apr;29(5):453–460. 28971712 10.1177/0956462417732649

[17] Tarka P, Nitsch-Osuch A, Gorynski P, et al. Epidemiology of pulmonary aspergillosis in hospitalized patients in Poland during 2009-2016. Adv Exp Med Biol. 2019;1160:73–80. 30919263 10.1007/5584_2019_347

[18] Escandón P, Lizarazo J, Agudelo CI, et al. Cryptococcosis in Colombia: compilation and analysis of data from laboratory-based surveillance. J Fungi (Basel). 2018;4(1):32. 10.3390/jof4010032 PMC5872335 29494502

[19] The Lancet Child Adolescent H. Cancer in the young: progress and priorities. Lancet Child Adolesc Health. 2018;2(3):157. 30169246 10.1016/S2352-4642(18)30041-5

[20] Chen KY, Ko SC, Hsueh PR, et al. Pulmonary fungal infection: emphasis on microbiological spectra, patient outcome, and prognostic factors. Chest. 2001;120(1):177–184. 11451835 10.1378/chest.120.1.177

[21] Peng L, Xu Z, Huo Z, et al. New insights into the clinical characteristics and prognostic factors of pulmonary fungal infections from a retrospective study in southwestern China. Infect Drug Resist. 2018;11:307–315. 29551904 10.2147/IDR.S157030 PMC5844258

[22] Liu Y, She D, Sun T, et al. A multicentre retrospective study of pulmonary mycosis clinically proven from 1998 to 2007. Zhonghua Jie He He Hu Xi Za Zhi. 2011;34(2):86–90. 21426723

[23] Vanittanakom N, Cooper CR, Fisher MC et al. Penicillium marneffei infection and recent advances in the epidemiology and molecular biology aspects. Clin Microbiol Rev 2006;19(9):5–110. 10.1128/CMR.19.1.95-110.2006 PMC1360277 16418525

[24] Gwinn M, MacCannell D, Armstrong GL. Next-generation sequencing of Infectious pathogens. Jama. 2019 Mar 5;321(9):893–894. 30763433 10.1001/jama.2018.21669 PMC6682455

[25] Simner PJ, Miller S, Carroll KC. Understanding the promises and hurdles of metagenomic next-generation sequencing as a diagnostic tool for infectious diseases. Clin Infect Dis. 2018;66(5):778–788. 29040428 10.1093/cid/cix881 PMC7108102

[26] Alanio A, Hauser PM, Lagrou K, et al. ECIL guidelines for the diagnosis of pneumocystis jirovecii pneumonia in patients with haematological malignancies and stem cell transplant recipients. J Antimicrob Chemother. 2016;71(9):2386–2396. 27550991 10.1093/jac/dkw156

[27] Schelenz S, Barnes RA, Barton RC, et al. British Society for medical mycology best practice recommendations for the diagnosis of serious fungal diseases. Lancet Infect Dis. 2015;15(4):461–474. 25771341 10.1016/S1473-3099(15)70006-X

[28] Donnelly JP, Chen SC, Kauffman CA, et al. Revision and update of the consensus definitions of invasive fungal disease from the european organization for research and Treatment of cancer and the mycoses study group education and research consortium. Clin Infect Dis. 2020;71(6):1367–1376. 31802125 10.1093/cid/ciz1008 PMC7486838

[29] Hage CA, Carmona EM, Epelbaum O, et al. Microbiological Laboratory testing in the diagnosis of fungal infections in pulmonary and critical care practice. An official American Thoracic Society clinical practice guideline. Am J Respir Crit Care Med. 2019;200(5):535–550. 31469325 10.1164/rccm.201906-1185ST PMC6727169

[30] Groll AH, Shah PM, Mentzel C, et al. Trends in the postmortem epidemiology of invasive fungal infections at a university hospital. J Infect. 1996;33(1):23–32. 8842991 10.1016/s0163-4453(96)92700-0

[31] Dimopoulos G, Frantzeskaki F, Poulakou G, et al. Invasive aspergillosis in the intensive care unit. Ann N Y Acad Sci. 2012 Dec;1272:31–39. 23231712 10.1111/j.1749-6632.2012.06805.x

[32] Schauwvlieghe AFAD, Rijnders BJA, Philips N, et al. Invasive aspergillosis in patients admitted to the intensive care unit with severe influenza: a retrospective cohort study. Lancet Respir Med. 2018;6(10):782–792. 30076119 10.1016/S2213-2600(18)30274-1

[33] Sass G, Nazik H, Penner J, et al. Aspergillus-Pseudomonas interaction, relevant to competition in airways. Med Mycol. 2019;57((Supplement_2)):S228–S232. 30816973 10.1093/mmy/myy087

[34] Briard B, Mislin GLA, Latgé J-P, et al. Interactions between Aspergillus fumigatus and pulmonary bacteria: current State of the field, New data, and future perspective. J Fungi (Basel). 2019;5(2):48. 10.3390/jof5020048 PMC6617096 31212791

[35] Briard B, Heddergott C, Latgé J-P. Volatile compounds emitted by Pseudomonas aeruginosa stimulate growth of the fungal pathogen Aspergillus fumigatus. mBio. 2016;7(2):e00219. 26980832 10.1128/mBio.00219-16 PMC4807360

[36] Bulpa P, Dive A, Sibille Y. Invasive pulmonary aspergillosis in patients with chronic obstructive pulmonary disease. Eur Respir J. 2007;30(4):782–800. 17906086 10.1183/09031936.00062206

[37] Máiz L, Nieto R, Cantón R, et al. Fungi in bronchiectasis: a concise review. Int J Mol Sci. 2018;19(1):142. 10.3390/ijms19010142 PMC5796091 29300314

[38] Martin-Loeches I, Antonelli M, Cuenca-Estrella M, et al. ESICM/ESCMID task force on practical management of invasive candidiasis in critically ill patients. Intensive Care Med. 2019;45(6):789–805. 30911804 10.1007/s00134-019-05599-w

