# Supplementary figures and images for: Trends of pulmonary fungal infections from 2013 to 2019: an AI-based real-world observational study in Guangzhou, China
Source: Emerg Microbes Infect. 2021 Mar 13;10(1):450–60. doi: 10.1080/22221751.2021.1894902 (PMC7971272; doi:10.1080/22221751.2021.1894902)

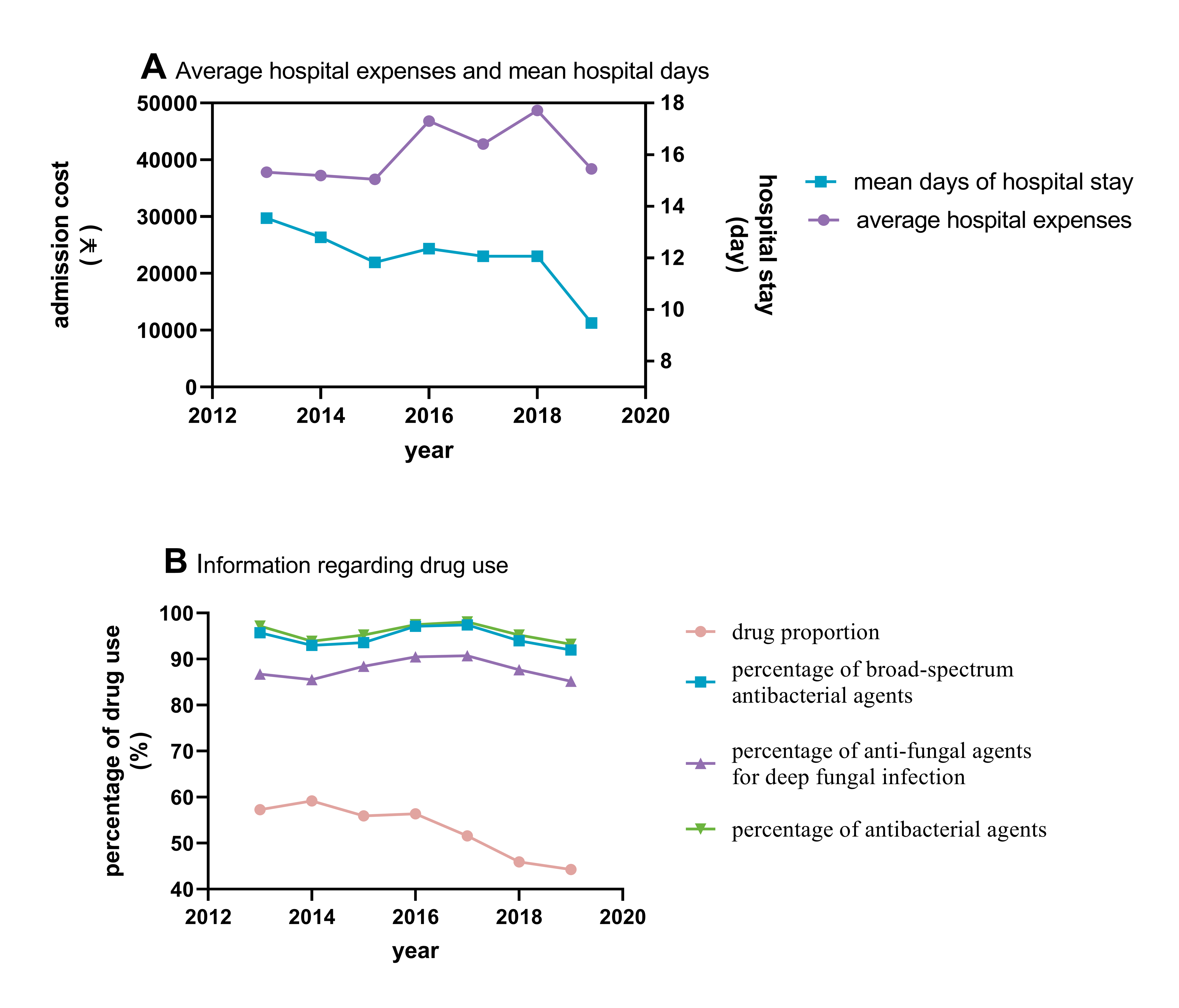

Supplement: eFigure1.tif [file TEMI_A_1894902_SM0270.tif]
