# Supplementary material for: Trends of pulmonary fungal infections from 2013 to 2019: an AI-based real-world observational study in Guangzhou, China
Source: Emerg Microbes Infect. 2021 Mar 13;10(1):450–60. doi: 10.1080/22221751.2021.1894902 (PMC7971272; doi:10.1080/22221751.2021.1894902)
Supplement: eTable.docx [file TEMI_A_1894902_SM0269.docx]

Supplemental Table 1: Number of PFI patients by clinical characteristics and assessment of trends over time

| **Number of PFI patients** | | | | |  |  |  |
| --- | --- | --- | --- | --- | --- | --- | --- |
|  | **2013** | **2014** | **2015** | **2016** | **2017** | **2018** | **2019** |
| **Total trends** |  |  |  |  |  |  |  |
| PFI inpatients | 155 | 154 | 174 | 203 | 226 | 365 | 367 |
| PFI outpatients | 112 | 167 | 173 | 232 | 286 | 444 | 470 |
| Inpatients with respiratory diseases | 5851 | 6372 | 6560 | 6793 | 7157 | 7896 | 8622 |
| Outpatients with respiratory diseases | 36426 | 39057 | 39310 | 41911 | 45907 | 51112 | 55410 |
| **Infections** |  |  |  |  |  |  |  |
| Pulmonary aspergillosis | 84 | 68 | 90 | 127 | 138 | 214 | 211 |
| Other pulmonary fungal disease | 47 | 58 | 40 | 44 | 37 | 74 | 72 |
| Pulmonary cryptococcosis | 23 | 27 | 36 | 31 | 39 | 65 | 59 |
| *Talaromyces marneffei* | 1 | 2 | 3 | 5 | 5 | 9 | 17 |
| Pulmonary mucormycosis | 1 | 8 | 6 | 3 | 10 | 4 | 8 |
| Pneumocystis pneumonia | 0 | 0 | 5 | 1 | 2 | 14 | 8 |
| Concurrent infection | 0 | 5 | 4 | 0 | 3 | 4 | 4 |
| Pulmonary candidiasis | 0 | 0 | 1 | 2 | 1 | 3 | 4 |
| Pulmonary coccidioidomycosis | 0 | 1 | 1 | 0 | 0 | 1 | 0 |
| **Sex (inpatients)** |  |  |  |  |  |  |  |
| Male | 106 | 105 | 123 | 149 | 164 | 252 | 243 |
| Female | 49 | 49 | 51 | 54 | 62 | 113 | 112 |
| **Sex (outpatients)** |  |  |  |  |  |  |  |
| Male | 65 | 102 | 110 | 149 | 185 | 287 | 302 |
| Female | 46 | 64 | 63 | 82 | 99 | 152 | 163 |
| **Age (inpatients)** |  |  |  |  |  |  |  |
| [0, 14] | 0 | 0 | 0 | 0 | 0 | 0 | 0 |
| (14, 30] | 2 | 7 | 9 | 18 | 20 | 24 | 34 |
| (30, 50] | 38 | 31 | 42 | 39 | 57 | 87 | 92 |
| (50, 70] | 68 | 63 | 78 | 95 | 103 | 192 | 169 |
| (70, ∞ ] | 47 | 53 | 45 | 51 | 46 | 62 | 72 |
| **Age (outpatients)** |  |  |  |  |  |  |  |
| [0, 14] | 1 | 1 | 0 | 0 | 0 | 1 | 0 |
| (14, 30] | 24 | 22 | 20 | 31 | 35 | 58 | 52 |
| (30, 50] | 36 | 59 | 70 | 80 | 97 | 138 | 154 |
| (50, 70] | 37 | 66 | 67 | 99 | 125 | 192 | 213 |
| (70, ∞ ] | 14 | 19 | 16 | 22 | 29 | 55 | 51 |
| **Underlying disease** |  |  |  |  |  |  |  |
| Pulmonary infection | 38 | 46 | 30 | 49 | 51 | 122 | 134 |
| Bronchiectasis | 39 | 26 | 36 | 55 | 60 | 93 | 81 |
| COPD | 29 | 33 | 30 | 50 | 51 | 91 | 57 |
| Diseases requiring mechanical ventilation | 29 | 21 | 18 | 40 | 36 | 79 | 87 |
| Hypertension | 31 | 32 | 35 | 40 | 43 | 72 | 49 |
| Diabetes | 25 | 33 | 31 | 37 | 40 | 49 | 46 |
| Tumor diseases | 14 | 18 | 24 | 30 | 23 | 71 | 45 |
| CTD | 20 | 14 | 13 | 21 | 39 | 40 | 25 |
| Hypoproteinemia | 9 | 14 | 15 | 16 | 11 | 36 | 36 |
| Diseases requiring invasive ventilation | 11 | 7 | 8 | 12 | 8 | 32 | 28 |
| **Adverse events** |  |  |  |  |  |  |  |
| Death | 2 | 0 | 1 | 2 | 2 | 3 | 4 |
| Invasive ventilation | 11 | 7 | 8 | 12 | 8 | 32 | 28 |
| ICU admission | 5 | 4 | 4 | 8 | 5 | 19 | 12 |
| Total | 12 | 7 | 8 | 14 | 11 | 37 | 30 |

COPD: chronic obstructive pulmonary disease. CTD: Connective tissue disease.

| Supplemental Table2: Number of PFIs patients with underlying diseases | | | | | | |  |  |
| --- | --- | --- | --- | --- | --- | --- | --- | --- |
|  | **2013** | **2014** | **2015** | **2016** | **2017** | **2018** | **2019** | **total** |
| Pulmonary infection | 38 | 46 | 30 | 49 | 51 | 122 | 134 | 470 |
| Bronchiectasis | 39 | 26 | 36 | 55 | 60 | 93 | 81 | 390 |
| COPD | 29 | 33 | 30 | 50 | 51 | 91 | 57 | 341 |
| Diseases requiring mechanical ventilation | 29 | 21 | 18 | 40 | 36 | 79 | 87 | 310 |
| Hypertension | 31 | 32 | 35 | 40 | 43 | 72 | 49 | 302 |
| Diabetes | 25 | 33 | 31 | 37 | 40 | 49 | 46 | 261 |
| Tumor diseases | 14 | 18 | 24 | 30 | 23 | 71 | 45 | 225 |
| CTD | 20 | 14 | 13 | 21 | 39 | 40 | 25 | 172 |
| Hypoproteinemia | 9 | 14 | 15 | 16 | 11 | 36 | 36 | 137 |
| Diseases requiring invasive ventilation | 11 | 7 | 8 | 12 | 8 | 32 | 28 | 106 |
| Heart failure | 10 | 13 | 7 | 15 | 9 | 23 | 11 | 88 |
| Anemia | 3 | 6 | 14 | 10 | 6 | 30 | 16 | 85 |
| pulmonary heart disease | 9 | 9 | 9 | 12 | 10 | 21 | 14 | 84 |
| coronary heart disease | 7 | 11 | 11 | 16 | 11 | 12 | 8 | 76 |
| Tuberculosis | 6 | 12 | 4 | 6 | 9 | 18 | 14 | 69 |
| Thrombosis | 6 | 3 | 9 | 11 | 8 | 13 | 10 | 60 |
| Asthma | 8 | 5 | 4 | 4 | 7 | 8 | 10 | 46 |
| Pneumothorax | 2 | 2 | 3 | 8 | 9 | 11 | 7 | 42 |
| Ankylosing spondylitis | 2 | 0 | 4 | 5 | 8 | 9 | 3 | 31 |
| Renal Failure | 2 | 3 | 3 | 5 | 2 | 10 | 5 | 30 |
| Heart Valve Disease | 4 | 1 | 2 | 2 | 2 | 9 | 8 | 28 |
| Rheumatoid arthritis | 3 | 0 | 0 | 4 | 6 | 6 | 4 | 23 |
| Lymphoma | 2 | 2 | 2 | 2 | 3 | 4 | 8 | 23 |
| Post-organ transplantation | 0 | 1 | 1 | 4 | 4 | 9 | 4 | 23 |
| Systemic lupus erythematosus | 0 | 1 | 0 | 5 | 5 | 6 | 5 | 22 |
| Pulmonary emphysema | 2 | 2 | 5 | 3 | 3 | 3 | 2 | 20 |
| Chronic kidney disease | 0 | 2 | 2 | 3 | 2 | 5 | 5 | 19 |
| Leukemia | 0 | 2 | 4 | 1 | 3 | 6 | 2 | 18 |
| Pulmonary embolism | 1 | 1 | 3 | 1 | 1 | 4 | 6 | 17 |
| Acute respiratory distress syndrome | 2 | 0 | 0 | 2 | 1 | 2 | 9 | 16 |
| Liver cirrhosis | 0 | 2 | 1 | 2 | 3 | 5 | 0 | 13 |
| hyperthyroidism | 1 | 1 | 1 | 3 | 2 | 3 | 1 | 12 |
| Lung abscess without pneumonia | 2 | 2 | 1 | 3 | 0 | 3 | 1 | 12 |
| Post-stem cell transplantation | 0 | 1 | 1 | 1 | 1 | 4 | 3 | 11 |
| Angina pectoris | 4 | 4 | 0 | 1 | 1 | 0 | 0 | 10 |
| Sjogrensyndrome | 1 | 2 | 2 | 2 | 1 | 1 | 1 | 10 |
| AIDS | 0 | 0 | 3 | 2 | 3 | 1 | 0 | 9 |
| Chronic heart failure | 1 | 1 | 0 | 1 | 2 | 3 | 0 | 8 |
| Hypertensive heart disease | 1 | 0 | 1 | 0 | 1 | 4 | 0 | 7 |
| host versus graft reaction | 0 | 0 | 1 | 1 | 1 | 1 | 3 | 7 |
| pulmonaryatelectasis | 0 | 2 | 0 | 3 | 1 | 0 | 0 | 6 |
| Rheumatic heart disease | 0 | 1 | 0 | 1 | 0 | 2 | 1 | 5 |
| sarcoidosis | 0 | 0 | 1 | 1 | 2 | 0 | 0 | 4 |
| Congenital heart disease | 0 | 1 | 0 | 1 | 2 | 0 | 0 | 4 |
| Myocardial infarction | 1 | 0 | 2 | 0 | 0 | 0 | 1 | 4 |
| Liver Failure | 0 | 0 | 0 | 0 | 0 | 4 | 0 | 4 |
| Silicosis | 0 | 0 | 0 | 0 | 1 | 1 | 1 | 3 |
| Influenza virus pneumonia | 0 | 0 | 0 | 0 | 0 | 1 | 2 | 3 |
| Dilated cardiomyopathy | 1 | 0 | 0 | 0 | 0 | 1 | 0 | 2 |
| Myelodysplastic syndrome | 0 | 0 | 0 | 1 | 0 | 0 | 1 | 2 |
| Sepsis | 2 | 0 | 0 | 0 | 0 | 0 | 0 | 2 |
| Acute Respiratory Failure | 1 | 0 | 0 | 0 | 1 | 0 | 0 | 2 |
| Myeloma | 0 | 0 | 0 | 0 | 0 | 1 | 1 | 2 |
| Multiple myeloma | 0 | 0 | 0 | 0 | 0 | 0 | 1 | 1 |
| Biliary ductitis | 0 | 1 | 0 | 0 | 0 | 0 | 0 | 1 |
| Acute pancreatitis | 0 | 0 | 0 | 0 | 1 | 0 | 0 | 1 |
| Subacute bacterial endocarditis | 0 | 0 | 0 | 0 | 0 | 0 | 0 | 0 |
| Ischemic cardiomyopathy | 0 | 0 | 0 | 0 | 0 | 0 | 0 | 0 |
| Immune thrombocytopenic purpura | 0 | 0 | 0 | 0 | 0 | 0 | 0 | 0 |
| Foot skin infections | 0 | 0 | 0 | 0 | 0 | 0 | 0 | 0 |
| Burns of body parts | 0 | 0 | 0 | 0 | 0 | 0 | 0 | 0 |
| Pregnancy | 0 | 0 | 0 | 0 | 0 | 0 | 0 | 0 |
| Thrombocytopenic purpura | 0 | 0 | 0 | 0 | 0 | 0 | 0 | 0 |
| Aplastic anemia | 0 | 0 | 0 | 0 | 0 | 0 | 0 | 0 |
| Neutropenia | 0 | 0 | 0 | 0 | 0 | 0 | 0 | 0 |
| Adult Still's disease | 0 | 0 | 0 | 0 | 0 | 0 | 0 | 0 |
| Overlap syndrome | 0 | 0 | 0 | 0 | 0 | 0 | 0 | 0 |

COPD: chronic obstructive pulmonary disease. CTD: Connective tissue disease.

Supplemental Table 3: Percentages of infections in PFIs patients.

| Infection | **Annual Percentage** | | | | | | | | **Percentage change  in annual incidence (95%Cl)*** | **P value** |
| --- | --- | --- | --- | --- | --- | --- | --- | --- | --- | --- |
|  | 2013 | | 2014 | 2015 | 2016 | 2017 | 2018 | 2019 |  |  |
| Pulmonary aspergillosis | | 54.19%(84) | 44.16%(68) | 51.72%(90) | 62.56%(127) | 61.06%(138) | 58.63%(214) | 57.49%(211) | 1.4(-0.8 to 3.6) | 0.165 |
| Other pulmonary fungal diseases | | 30.32%(47) | 37.66%(58) | 22.99%(40) | 21.67%(44) | 16.37%(37) | 20.27%(74) | 19.62%(72) | -4.5(-8.5 to -0.5) | 0.035 |
| Pulmonary cryptococccosis | | 14.84%(23) | 17.53%(27) | 20.69%(36) | 15.27%(31) | 17.26%(39) | 17.81%(65) | 16.08%(59) | 0.1(-2.4 to 2.7) | 0.895 |
| Talaromyces Marneffei | | 0.65%(1) | 1.30%(2) | 1.72%(3) | 2.46%(5) | 2.21%(5) | 2.47%(9) | 4.63%(17) | 11.5(6.4 to 16.7) | 0.002 |
| Pulmonary mucormycosis | | 0.65%(1) | 5.19%(8) | 3.45%(6) | 1.48%(3) | 4.42%(10) | 1.10%(4) | 2.18%(8) | 1.2(-16.5 to 19.0) | 0.866 |
| Pneumocystis pneumonia | | 0.00%(0) | 0.00%(0) | 2.87%(5) | 0.49%(1) | 0.88%(2) | 3.84%(14) | 2.18%(8) | 6.5(-35.1 to 48.1) | 0.652 |
| Concurrent Infection | | 0.00%(0) | 3.25%(5) | 2.30%(4) | 0.00%(0) | 1.33%(3) | 1.10%(4) | 1.09%(4) | -10.0(-14.2 to -5.7) | 0.005 |
| Pulmonary candidiasis | | 0.00%(0) | 0.00%(0) | 0.57%(1) | 0.99%(2) | 0.44%(1) | 0.82%(3) | 1.09%(4) | 0.048(-0.122 to 0.217) | 0.436 |
| Pulmonary coccidioidomycosis | | 0.00%(0) | 0.65%(1) | 0.57%(1) | 0.00%(0) | 0.00%(0) | 0.27%(1) | 0.00%(0) | -0.097(-0.235 to 0.041) | 0.071 |

The data represent the percentage of patients with a particular type of PFI among all PFI inpatients unless otherwise stated. * Estimated using linear regression analysis of the log of the annual percentage.

| Adverse event | **Annual Incidence** | | | | | | | **Percentage change  in annual incidence (95%Cl)*** | **P value** |
| --- | --- | --- | --- | --- | --- | --- | --- | --- | --- |
|  | 2013 | 2014 | 2015 | 2016 | 2017 | 2018 | 2019 |  |  |
| total | 7.74(12) | 4.55(7) | 4.60(8) | 6.90(14) | 4.87(11) | 10.14(37) | 8.17(30) | 0.5(-0.6 to 1.5) | 0.3 |
| Death | 1.29(2) | 0.00(0) | 0.57(1) | 0.99(2) | 0.88(2) | 0.82(3) | 1.09(4) | 0.04(-0.2 to 0.3) | 0.591 |
| Invasive ventilation | 7.10(11) | 4.55(7) | 4.60(8) | 5.91(12) | 3.54(8) | 8.77(32) | 7.63(28) | 0.3(-0.6 to 1.3) | 0.421 |
| ICU | 3.23(5) | 2.60(4) | 2.30(4) | 3.94(8) | 2.21(5) | 5.21(19) | 3.27(12) | 0.2(-0.3 to 0.7) | 0.395 |

Supplemental Table 4: Incidence of PFIs patients by adverse event.

The data represent the incidence per 100 PFI patients unless otherwise stated. * Estimated using a linear regression analysis of the log of the annual incidence.
